# The long noncoding RNA CASC9 regulates migration and invasion in esophageal cancer

**DOI:** 10.1002/cam4.770

**Published:** 2016-07-19

**Authors:** Zhiwen Pan, Weimin Mao, Yejiang Bao, Min Zhang, Xinhua Su, Xiaohong Xu

**Affiliations:** ^1^ Department of Clinical LaboratoryZhejiang Province Cancer HospitalHangzhouZhejiang310000China; ^2^ Zhejiang Key Laboratory of Diagnosis & Treatment Technology on Thoracic Oncology (Lung and Esophagus)HangzhouZhejiang310000China

**Keywords:** CASC9, esophageal cancer, invasion, long noncoding RNA, migration

## Abstract

The objective of the study was to investigate the expression and functions of CASC9 in esophageal squamous cell carcinoma (ESCC). Long noncoding RNAs (lncRNAs) upregulated in ESCC tissues were detected by RNA sequencing. Expression of CASC9 was determined from clinical samples and cell lines by qRT‐PCR. The effects of CASC9 knockdown on migration and invasion were evaluated by wound healing assay, cell migration and invasion assays in vitro. We found that the lncRNA, CASC9, was markedly upregulated in ESCC tissues. Furthermore, knockdown of CASC9 significantly suppressed cell migration and invasion in vitro. Furthermore, enhanced CASC9 expression level was correlated with differentiation. The results indicated that CASC9 is significantly upregulated in ESCC tissues and may represent a new marker of poor prognosis and a potential therapeutic target for esophageal cancer intervention.

## Introduction

Esophageal cancer (EC) is one of the leading aggressive malignancies worldwide and ranks as one of the top five deadliest cancers in China, while esophageal squamous cell carcinoma (ESCC) accounts for the most prevalent histopathologic type 1. Based on IARC statistics, there are 456,000 new cases (3.2% of the total) and 400,000 deaths (4.9% of the total) in 2012 2. The incidence and mortality of EC in China were 22.4/100,000 and 16.77/100,000, respectively 3. Despite the recent advances in EC treatment, the prognosis is still unfavorable, making it essential for uncovering the underlying mechanisms of EC for therapy improvement. Therefore, better understanding of the pathogenesis and identification of the molecular alterations is essential for the development of useful indicators that aid novel effective therapies for EC.

In 2002, full‐length mouse complementary DNA sequencing resulted in the identification of noncoding RNA for the first time, which indicated that noncoding RNA is a major component of the transcriptome 4. Noncoding RNAs, which are known as long noncoding RNAs (lncRNAs), are RNA molecules longer than 200 nucleotides that are not translated into proteins 5. Furthermore, more and more evidences have shown that lncRNAs are key regulators in several biological processes, such as embryonic growth, cell proliferation, differentiation, transcriptional, and post‐transcriptional regulators of gene activity 6, 7. Accumulating data have also indicated that the dysregulation of lncRNAs may play an important functional role in diverse human cancers, including esophageal cancer 8. List of all known lncRNAs, H19 is the first identified lncRNA which possesses the function of gene imprinting and is described as a tumor suppressor, and more recent analysis show that H19 expression is reactivated in breast, endometrial, lung, cervical, esophageal, and bladder tumors 9, 10. Yang et al. have shown that higher levels of lncRNA HNF1A‐AS1 expression were observed in esophageal adenocarcinoma, and they described the effects of this lncRNA on cell proliferation, cell cycle regulation, migration, and invasion. They also proved that lncRNA H19 may represent a downstream effector of HNF1A‐AS1. These results indicated that the dysregulation of lncRNAs could participate in tumor progression 11, 12.

Cancer susceptibility candidate 9 (CASC9), mapped to human chromosome 8q21.11, was originally identified as an esophageal squamous cell carcinoma‐associated lncRNA. Following studies have documented that CASC9 is highly expressed in 65% of ESCC samples relative to adjacent normal tissues, its higher expression frequency in ESCC is compatible with onco‐lncRNA HOTAIR expression in ESCC 13. Furthermore, the knockdown of CASC9 expression increases apoptosis and decreased invasive capacity significantly in vitro 14. These results suggest that CASC9 may contribute to inhibit cell death program and facilitate the invasion and metastasis, however, the detail mechanisms remain to be largely undefined.

In the current study, a next‐generation sequencing analysis of human esophageal tissues has uncovered marked upregulation of the lncRNA CASC9 in ESCC tissue relative to normal esophagus. Then, quantitative reverse transcriptase PCR (qRT‐PCR) was performed to investigate the expression of CASC9 in 44 cancer tissues and five ESCC cell lines. We further discovered that siRNA‐mediated knockdown of CASC9 results in diminished cell migration and invasion in ESCC cells. Taken together, these findings suggest that ESCC participates as a noncoding oncogene in esophageal tumorigenesis.

## Materials and Methods

### Tissue collection

ESCC and corresponding normal esophageal epithelial tissues were obtained from 44 patients who underwent surgery resection at Zhejiang Cancer Hospital, Hangzhou, Zhejiang, China. Clinical information was collected from medical records. All persons gave their informed consent prior to their inclusion in the study. No patients had received local or systemic treatment before surgery. All specimens were snap‐frozen by liquid nitrogen and stored at −80°C until use.

### Next‐generation RNA sequencing

RNA sequencing (RNA‐seq) of five paired esophageal tissues was carried out using the Illumina HiSeq‐2500 sequencer (paired‐end reads) platform.

### Cell lines and culture conditions

Five esophageal squamous cell carcinoma cell lines (TE1, Kyse150, EC109, EC9706, and EC1) and human embryonic kidney 293T were obtained from the Cell Bank of the Chinese Academy of Sciences (Shanghai, China). All cells were cultured in DMEM high medium (Hyclone, Logan, Utah, USA) supplemented with 10% fetal bovine serum (10% FBS), 100 U/mL penicillin, and 100 mg/mL streptomycin (Invitrogen, Shanghai, China), and maintained in a humidified incubator at 37°C with 5% CO_2_.

### RNA extraction and qRT‐PCR

The total RNA was isolated with Trizol reagent (Invitrogen, Carlsbad, CA) according to the manufacturer's protocols. The isolated RNA was reverse‐transcribed into cDNA using ReverTra Ace qPCR RT kit (Toyobo, Shanghai, People's Republic of China). Briefly, a total of 1000 ng RNA was used for the initial reverse transcription reaction.

Real‐time qRT‐PCR for mRNA was performed with KAPA SYBR FAST qPCR kit (Kapa SYBR, Wilmington, Massachusetts, USA) according to the manufacturer's instruction. qRT‐PCR data collection was performed on an ABI 7500 apparatus (Applied Biosystems, Foster City, CA). The PCR reaction was conducted for 30 sec at 95°C, 40 cycles at 95°C for 30 sec, and 60°C for 30 sec. RNA 18S was carried out as endogenous control in each sample. The relative quantification of mRNA expression was calculated using the 2^−△△Ct^ method relative to 18S level. All of the qRT‐PCR reactions were replicated three times. The PCR primers for mRNA or 18S were as follows: CASC9 sense 5′‐AGATGAAGCCGGTACCTCAGAT‐3′, reverse 5′‐TCACTTTAAAGAGGGAGAGGAG‐3′; 18S sense 5′‐CAGCCACCCGAGATTGAGCA‐3′, reverse 5′‐TAGTAGCGACGGGCGGTGTG‐3′.

### Small interfering RNA transfection

Three small interfering RNAs (siRNAs) against CASC9 (si‐CASC9) at different sites and one negative control (si‐NC) with no definite target were employed and synthesized by Tuoran (Shanghai, China). Cells were seeded on six‐well plates at a density of 1.5 × 10^5^/well overnight, and then transfected with siRNA or the negative control at a final concentration of 75 nmol/L using lipofectamine 2000 (Invitrogen, USA). The interfering efficiency was determined by qRT‐PCR 48 h after transfection, and the siRNAs with silencing efficacy of more than 70% were selected for further experiments.

### Wound healing assay

The cells were seeded in six‐well plate until the cells reached to 90% confluency. The confluent monolayers were scratched by a 200 *μ*L pipette tip to generate the wound. The debris and floating cells were removed through washing with PBS. The cells were cultured in the medium supplemented with 1% FBS for 48 h to allow wound healing. The photographic images were taken at different time points.

### Cell migration/invasion assay

At 24 h after transfection, cells in serum‐free media were seeded into the upper chamber for migration assays (8 *μ*m pore size, Millipore) and invasion assays with Matrigel (Sigma‐Aldrich, St Louis, MO, USA). The lower chambers were filled with media containing 15% FBS. After several hours of incubation at 37°C, the cells that had migrated or invaded through the membrane were fixed in paraformaldehyde and stained with 0.1% crystal violet (Sigma). The cells on the lower surface were photographed and five random fields were counted. Three independent experiments were performed.

### Statistical analysis

Statistical comparisons between two different groups were determined by Student's *t* test using GraphPad Prism 4.0 (Graphpad Software, La Jolla, CA) and chi‐square test was used for clinicopathological association analysis. The results were presented as means ± SEM. *P* < 0.05 was considered statistically significant.

## Results

### RNA‐seq analysis detects lncRNAs upregulated in ESCC

**Figure 1:**
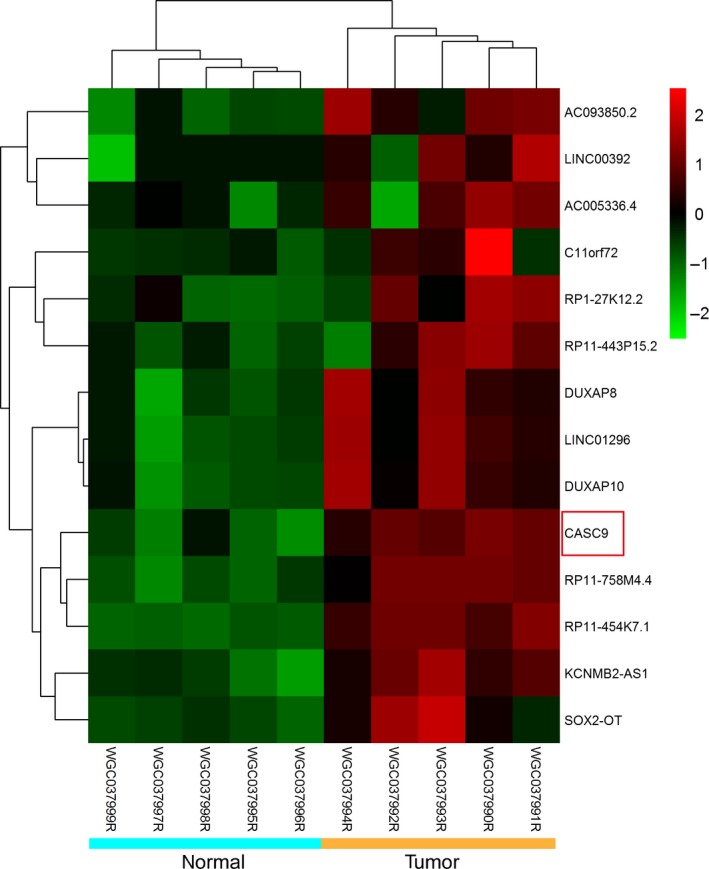
Results of cluster analysis for the 14 differentially expressed lncRNAs of five matched esophageal squamous cell carcinoma (ESCC) tissue pairs according to RNA‐seq.

In order to identify novel oncogene in ESCC, RNA‐seq of five matched ESCC tissue pairs was carried out. The RNA‐seq analysis detected 12 lncRNAs that showed relative high expression in ESCC tissues (fold change >5) (Fig. 1). We prioritized lncRNAs that showed the highest expression in ESCC and identified three lncRNAs (LINC00392, RP1‐27K12.2, and CASC9). Notably, it has been reported that CASC9 was highly expressed in ESCC tissues. However, the detail mechanisms remain to be largely undefined. Therefore, the selection of CASC9 appears to underscore the validity of our filtering approach.

### CASC9 is upregulated in ESCC tissues

**Figure 2:**
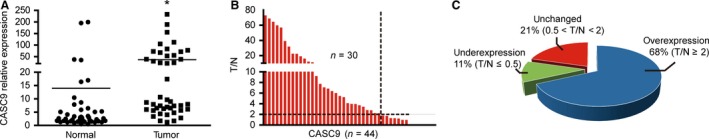
Relative CASC9 expression in esophageal squamous cell carcinoma (ESCC) tissues. (A) Relative expression of CASC9 in ESCC tissues (*n* = 44) in comparison with corresponding nontumor normal tissues (*n* = 44). CASC9 expression was examined by qRT‐PCR and normalized to 18S expression. (B) The ratio of CASC9 expression in human ESCC specimens and corresponding normal tissues (T/N). Forty‐four pairs of tissues were used for the assay, and the ratio was divided into two parts by T/N = 2 (black solid line). Thirty pairs had a ratio above twofold (left of the black dashed line), and the others had a ratio below twofold (right of the black dashed line). (C) The distribution of CASC9 expression in clinical specimens. The pie was divided into three parts by T/N = 0.5 and T/N = 2.

The level of CASC9 expression was determined in 44 paired esophageal cancer samples and adjacent, histologically normal tissues by qRT‐PCR, and normalized to 18S. CASC9 expression was significantly upregulated in cancerous tissues (*P* < 0.05) compared with normal counterparts (Fig. 2A). The results from tissues showed that CASC9 was overexpressed in 68% of the tumor specimens relative to normal tissues (Fig. 2B and C), indicating that the upregulation of CASC9 is associated with ESCC development.

### CASC9 expression in ESCC cell lines

**Figure 3:**
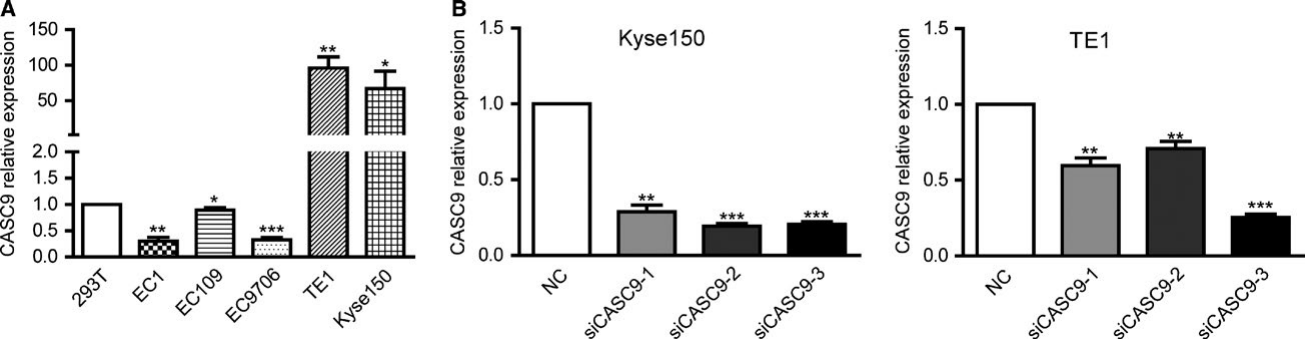
CASC9 expression in esophageal squamous cell carcinoma (ESCC) cells. (A) Expression level of CASC9 in ESCC cell lines (esophageal cancer [EC]1, EC109, EC9706, TE1, and Kyse150) compared with that of human embryonic kidney cell line 293T. (B) Expression level of CASC9 in Kyse150 and TE1 following treatment with siCASC9 or siNC *P<0.05; **P<0.01;***P<0.001.

To explore the role of CASC9 in the development of ESCC, we next performed qRT‐PCR analysis to assess CASC9 expression in ESCC cell lines. Compared with that in human embryonic kidney 293T cells, CASC9 expression was at a comparatively high level in two cell lines, including TE1 and Kyse150 (Fig. 3A). To downregulate endogenous CASC9 in ESCC growth, we silenced CASC9 expression in Kyse150 and TE1 by small interfering RNA. The siRNA which decreased CASC9 expression level by more than 70% was chosen for further experiments. At 48 h post‐transfection, CASC9 expression was knocked down by approximately 80% in Kyse150 and 75% in TE1 cells by siCASC9‐3 transfection when compared with the scrambled siRNA (Fig. 3B). Of these three siRNAs, siRNA3 was chosen for further assays of Kyse150 and TE1.

### CASC9 knockdown suppresses cell migration and invasion in vitro

**Figure 4:**
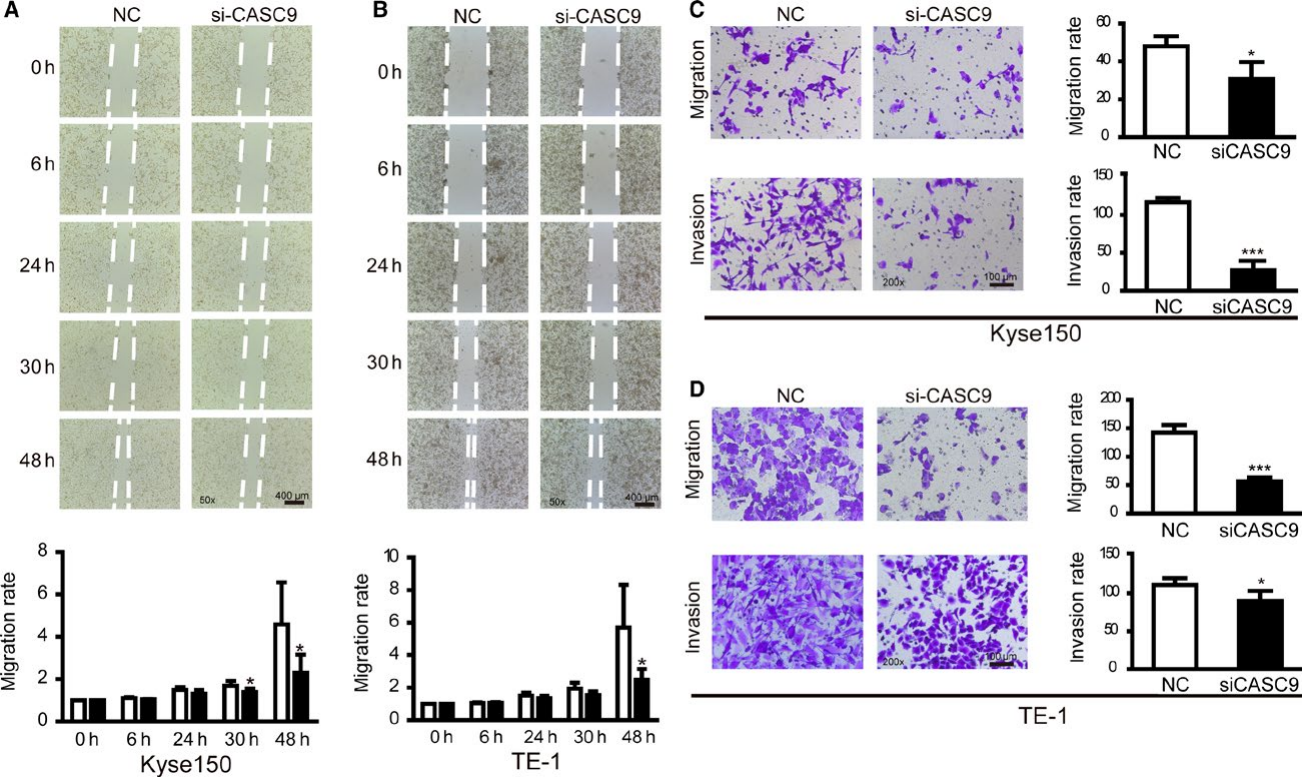
CASC9 knockdown suppressed cell migration and invasion in esophageal squamous cell carcinoma (ESCC) cells. (A and B) Wound healing assays were used to detect the motility in Kyse150 and TE‐1 transfected with siCASC9. (C and D) Transwell assays were conducted in Kyse150 and TE‐1 transfected with siCASC9 and quantitative results were illustrated for panels C and D. *P < 0.05, ***P < 0.001 versus control.

To determine whether the inhibition of CASC9 expression can suppress ESCC migration and invasion, cell wound healing and transwell assays were carried out to evaluate cancer cell migration and invasion. Wound healing assay showed that decreased CASC9 expression inhibited the cell motility in both Kyse150 and TE‐1 cells when compared with control groups (Fig. 4A and B). Downregulation of CASC9 expression also impaired the migration and invasion capacity of Kyse150 and TE‐1 by siRNA interference (Fig. 4C and D). Our results suggest that CASC9 could play a critical role in governing cell migration and invasion.

### The relationship between CASC9 expression and clinicopathological factors in patients with esophageal cancer

We also investigated the association between CASC9 expression and clinicopathological factors of tumor. The results revealed that higher positive expression of CASC9 correlated with the differentiation (*P* < 0.001). However, there was no significant association among CASC9 expression and age, gender, tumor size, invasion, lymph node metastases, and TNM stage (Table 1).

**Table 1:** Relationship between CASC9 expression and clinicopathological features of ESCC

Characteristics	*n*	CASC9 expression		*P*
		Positive	Negative	
Gender				
Male	29	19	10	0.282
Female	13	11	2	
Age				
<60	23	17	6	0.742
≥60	19	13	6	
Tumor size (cm)				
<4	19	15	4	0.496
≥4	23	15	8	
Differentiation				
Well	10	2	8	<0.001
Moderate	23	19	4	
Poor	9	9	0	
Tumor invasion (T)				
T1–T2	7	5	2	1.00
T3–T4	35	25	10	
Lymph node metastasis (N)				
Absent	18	13	5	1.00
Present	24	17	7	
TNM stage				
I + II	22	15	7	0.738
III + IV	20	15	5	

## Discussion

It has become increasingly apparent that mammalian genomes encode numerous lncRNAs, which are more than 200 nucleotides in length with limited protein‐coding capacity 15. In recent years, studies have shown that 18% of the protein‐coding genes that produce lncRNAs are associated with cancer, whereas only 9% of all human protein‐coding genes are associated with cancer 16. Due to their great importance in the regulation of gene expression, it has been widely accepted that lncRNAs are involved in multiple cellular functions including proliferation, apoptosis, and differentiation, thus have been implemented in diverse physiological and pathological processes ranging from development to cancer 17. Therefore, the identification and investigation of cancer‐associated lncRNAs is critical for understanding the roles of lncRNAs in cancer progression and may be important for novel therapeutic targets.

CASC9 is a lncRNA identified by a next‐generation sequencing analysis in esophageal cancer which has been demonstrated overexpressed in ESCC through bioinformatics analyses in several researches 14, 18, 19. One study reported that dysregulation of CASC9 was associated with apoptosis and invasion, but none provided data of clinical tissues. In the present study, we have investigated lncRNA CASC9 expression by qRT‐PCR assay in 44 paired ESCC tissues. The results indicated that expression of CASC9 in ESCC tissues was significantly higher than those in adjacent normal tissues. Specifically, CASC9 expression was significantly higher in poor differentiation tumors. Furthermore, CASC9 knockdown significantly inhibited esophageal cancer cell migration and invasion in vitro. In conclusion, these results demonstrated that CASC9 might be a novel prognostic indicator in ESCC and may be a potential target for diagnosis and gene therapy.

In summary, this study identifies CASC9 as a novel potential oncogene in ESCC that acts by cell migration and invasion. However, downstream regulator involved in CASC9‐mediated cell migration and invasion in ESCC cells remains to be unclarified. Thus, much more work is still required to determine the detailed mechanisms it functions in ESCC and the potentiality of CASC9 as a therapeutic target for ESCC.

## Conflict of Interest

None declared.
